# Evidence of and deaths from malaria and severe pneumonia co-infections in malaria-endemic areas: a systematic review and meta-analysis

**DOI:** 10.1038/s41598-022-22151-x

**Published:** 2022-10-15

**Authors:** Wanida Mala, Polrat Wilairatana, Giovanni De Jesus Milanez, Frederick Ramirez Masangkay, Kwuntida Uthaisar Kotepui, Manas Kotepui

**Affiliations:** 1 Medical Technology, School of Allied Health Sciences, Walailak University, Tha Sala, Nakhon Si Thammarat, Thailand; 2 Center of Excellence Research for Melioidosis and Microorganisms, Walailak University, Nakhon Si Thammarat, 80160 Thailand; 3 Department of Clinical Tropical Medicine, Faculty of Tropical Medicine, Mahidol University, Bangkok, 10400 Thailand; 4 Department of Medical Technology, Faculty of Pharmacy, Royal and Pontifical University of Santo Tomas, Manila, Philippines

**Keywords:** Malaria, Viral infection

## Abstract

Malaria and pneumonia are the leading causes of childhood mortality in children under 5 years of age. Nevertheless, the proportions and deaths of malaria co-infection among patients with severe pneumonia, particularly in children under 5 years of age, and characteristics of co-infection remain poorly explored. Hence, the present study aimed to collate the evidence of malaria among patients with severe pneumonia, severe pneumonia among patients with malaria, and the proportion of deaths among patients with co-infections. Potentially relevant studies were searched in six databases including PubMed, Scopus, Web of Science, Embase, Ovid, and MEDLINE to identify studies on malaria and severe pneumonia co-infections that were published until 21 July 2022 with a restriction for the non-English language but no restriction for the publication year. The quality of the included studies was determined using the Strengthening the Reporting of Observational Studies in Epidemiology (STROBE). The pooled estimates, including the pooled proportion of malaria among patients with severe pneumonia, and the proportion of deaths among patients with co-infections, were estimated by the random-effects model. Of the 4094 studies examined, 11 studies that met the eligibility criteria were included in the review. Meta-analysis results showed that the proportion of malaria (2162 cases) among patients with severe pneumonia (9738 cases) was 19% (95% CI 12–26%, I^2^: 98.79%, 11 studies). The proportion of severe pneumonia (546 cases) among patients with malaria (10,325 cases) was 20% (95% CI 0–40%, I^2^: 99.48%, 4 studies). The proportion of deaths among patients with co-infection was 13% (95% CI 2–23%, I^2^: 85.1%, 3 studies). In conclusion, nearly one-fifth of patients with severe pneumonia have malaria, one-fifth of patients with malaria have severe pneumonia, and about 13% of co-infections lead to deaths. This information raised the clinical importance of diagnosis and management of concurrent infections. Patients with severe pneumonia should be investigated for malaria, and vice versa. Detection of co-infections might provide the information to inform the physician to manage and cure co-infected patients who live in areas where both diseases were endemic.

## Introduction

Malaria, pneumonia, and malnutrition are the leading causes of childhood mortality in children under 5 years of age^1–4^. For malaria, 229 million cases and 409,000 deaths were estimated worldwide in 2019^5^. More than half of malaria cases globally were reported from Nigeria (27%), the Democratic Republic of the Congo (12%), Uganda (5%), Mozambique (4%), and Nigeria (3%)^5^. Meanwhile, more than half of malaria deaths globally were reported from Nigeria (23%), the Democratic Republic of the Congo (11%), the United Republic of Tanzania (5%), Mozambique (4%), Nigeria (4%) and Burkina Faso (4%)^5^. For pneumonia, it was reported as 14% of all deaths of children under 5 years old with a total number of 740,180 deaths in 2019^6^. The high burden of cases and deaths from malaria and pneumonia were found in malaria‐endemic regions, particularly in low-income countries^1^.

In areas where malaria is endemic, the clinical manifestations of malaria might overlap with those of bacteremia, meningitis, or pneumonia^7–10^. In resource-limited settings where diagnostic tools for pneumonia and malaria may not be accessible or available, distinguishing between pneumonia and malaria in children is challenging as these two diseases present with some overlapping clinical signs and symptoms^11^. A study suggested that overlapping clinical features of severe malaria and severe pneumonia included fever, respiratory distress, and impaired consciousness^12^. Dehydration, lower packed cell volume (PCV), and hyperbilirubinemia were suggested to be the predictive markers of severe malaria. Meanwhile, hypoxemia, preadmission antibiotic treatment, chest signs, and higher leukocyte counts were indicated to be the predictive markers of severe pneumonia^11^. The risks of underestimating the overlapping clinical symptoms of malaria and severe pneumonia could lead to missed treatment for both diseases^13,14^.

The previous study showed that among children admitted to the hospital with severe pneumonia, 19% of them had malaria^15^. Another study suggested that although the actual overlap of severe pneumonia and malaria was uncommon, no reliable tools could distinguish between these two diseases^11^. Previous studies also indicated that children were positive for malaria in endemic areas, and children with respiratory signs, both pneumonia and malaria should be treated^13,14^. Although the differentiation of malaria from severe pneumonia had been attempted in literature^11,12,16^, the prevalence and probability of co-infection between the two diseases remain poorly explored. Malaria and severe pneumonia comorbidity are of wide interest to clinicians, public health professionals, and policymakers in considering the appropriate time to add antibiotics for the treatment of pneumonia in children with severe malaria. Hence, the present study aimed to collate the evidence of malaria among patients with severe pneumonia, severe pneumonia among patients with malaria, and the proportion of deaths among patients with co-infections.

## Methods

### Protocol and search strategy

The systematic review was registered at PROSPERO (ID: CRD42021290750). The systematic review and meta-analyses adhered to Preferred Reporting Items for Systematic Reviews and Meta-Analyses (PRISMA) guidelines^17^. Potentially relevant studies were searched in six databases including PubMed, Scopus, Web of Science, Embase, Ovid, and MEDLINE to identify malaria and severe pneumonia co-infection in children admitted to hospitals published until 21 July 2022. There was a restriction for non-English language but no restriction for the publication year. The combination of search terms “(Malaria OR Plasmodium)” AND (Pneumonias OR Pneumonia OR “Lung Inflammation” OR “Lung Inflammations” OR “Pneumonitis” OR “Pneumonitides” OR “Pulmonary Inflammation” OR “Pulmonary Inflammations”) AND (severe OR complicated)” were used to identify relevant studies (Table S1).

### Definitions

1. Malaria: The occurrence of malaria infection in a person in whom the presence of malaria parasites in the blood has been confirmed by a diagnostic test such as microscopy, serology, or rapid diagnostic testing (RDT)^18^.
2. Severe pneumonia: Children who present with any general danger sign or chest drawing or stridor in calm child according to the Integrated Management of Childhood Illness (IMCI) definition which is developed by the World Health Organization (WHO)^19^.
3. Malaria and severe pneumonia co-infections: Children who present with both malaria and severe pneumonia.

### Eligibility criteria

PICo was applied for eligibility criteria; P: participants hospitalized for severe pneumonia or malaria, I: outcome of interest was co-infections, Co: context was endemic regions for malaria. Studies that reported the co-infection of malaria and severe pneumonia in participants were examined for inclusion. Study designs could be cross-sectional studies, cohort studies, case–control studies, retrospective or prospective observational studies, or clinical trials. Short reports, case series, case reports, letters to the editor, review, systematic review, comments, and opinions were excluded.

### Study selection and data extraction

Two authors (WM, MK) used the predetermined eligibility criteria to screen titles, abstracts, and full texts of relevant studies. Meanwhile, a third author (PW) adjudicated if the first two authors did not agree. After relevant studies were selected, the following data were extracted from each study to the pilot excel datasheet; first author, publication year, study sites, characteristics of participants, age, sex (male percentage), number of malaria and severe pneumonia co-infection, *Plasmodium* spp., number of malaria, number of severe pneumonia, clinical outcomes of co-infection, number of deaths among patients with co-infection, diagnostic methods for malaria, and diagnostic methods for severe pneumonia. Similar to study selection, two authors (WM, MK) participated in data extraction, and the third author (PW) adjudicated if the first two authors did not agree.

### Quality of the included studies

The quality of the included studies was determined using the Strengthening the Reporting of Observational Studies in Epidemiology (STROBE) which provided the checklist items for observational studies^20^. A total of 22 items (total score = 22) were used to assess the overall quality of the included studies. We also assessed the methodological quality of the included studies based on nine items including study design, setting, participants, variables, data sources/measurement, bias, study size, quantitative variables, and statistical methods (total score = 9). Any study rated more than 75% indicated a high-quality study. Meanwhile, any study rated less than 75% showed moderate/low quality.

### Data analysis

Outcomes of interest were (1) the proportion of malaria among patients with severe pneumonia, (2) the proportion of severe pneumonia among patients with malaria, and (3) the proportion of deaths among patients with co-infection. The pooled estimates, including the pooled proportion of malaria among patients with severe pneumonia, the pooled proportion of severe pneumonia among patients with malaria, and the pooled proportion of deaths among patients with co-infection, were estimated by the random-effects model^21^. Results of the meta-analysis were shown as effect estimates, 95% confidence interval (CI), and weights in forest plots. The meta-regression analysis based on the methodological quality of the studies, publication year, country, study design, age groups, and diagnostic methods for malaria had been performed to test whether these parameters were the source (s) of heterogeneity of the pool proportion of the effect estimates or not. Subgroups analysis of covariates that were significant in the meta-regression analysis were performed to inspect the difference in the individual subgroup. Between-study heterogeneity was estimated using the I^2^ statistic to determine between-study heterogeneity of effect estimates. I^2^ statistic with < 50%, and 50–75%, and > 75% indicating moderate, substantial, and high heterogeneity^22^. Sensitivity analysis using the different diagnostic criteria for severe pneumonia was performed to determine the robustness of the meta-analysis results if some studies using non-standardized criteria for severe pneumonia confounded the effect size calculated by the random-effects model. Publication bias among the included studies was not performed due the meta-analyses were based on the prevalence data. All statistical analyses were carried out using Stata version 14 (StataCorp LLC, Texas, USA).

## Results

### Search results

**Figure 1:**
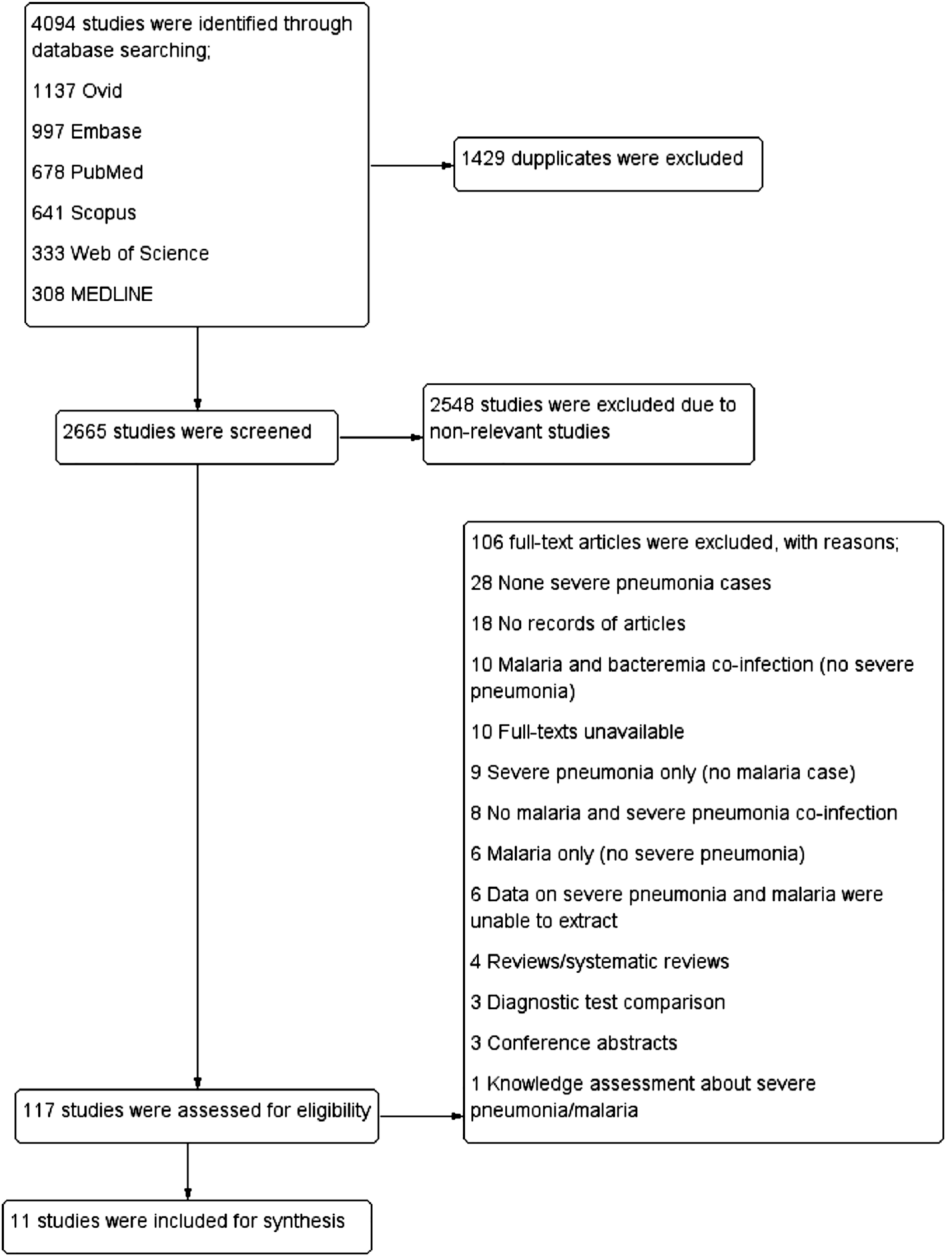
Flowchart for the study selection. Flowchart demonstrates the selection of potentially relevant studies.

A total of 4094 studies were identified from Ovid (1137 studies), Embase (997 studies), PubMed (678 studies), Scopus (641 studies), Web of Science (333 studies), and MEDLINE (308 studies). After 1429 duplicates were excluded, the remaining 2665 studies were screened for titles and abstracts, and 2548 non-relevant studies were excluded. Then the remaining 117 studies were assessed for eligibility, and 106 full-text articles were excluded, for specific reasons; 28 none severe pneumonia cases, 18 no records of articles, 10 malaria and bacteremia co-infection (no severe pneumonia), 10 full-texts unavailable, 9 severe pneumonia only (no malaria case), 8 no malaria and severe pneumonia co-infection, 6 malaria only (no severe pneumonia), 6 data on severe pneumonia and malaria were unable to extract, 4 review/systematic reviews, 3 diagnostic test comparison, 3 conference abstracts, and 1 with knowledge assessment about severe pneumonia/malaria. Finally, 11 studies^7,11,12,16,23–29^ met the eligibility criteria and were included in qualitative and quantitative syntheses (Fig. 1).

### Characteristics of the included studies

The 11 included studies were published between 2005 and 2022, and about half (54.5%) were published between 2011 and 2022. Most of the included studies were conducted in Mozambique (4, 36.4%)^11,15,27,30^, Tanzania (2, 18.2%)^16,29^, Kenya (2, 18.2%)^7,24^, Nigeria (1, 9%)^12^, Uganda (1, 9%)^26^, and Malawi (1, 9%)^25^. Most of the studies were prospective observational studies (7, 63.6%), prospective and retrospective observational studies (2, 18.2%), and retrospective observational studies (2, 18.2%). Most of the included studies did not report species of *Plasmodium* (9, 81.8%), meanwhile, two studies (18.2%)^24,27^ reported *P. falciparum*. Five studies (45.5%)^15,16,25–27^ reported deaths among co-infected patients, while others did not. The microscopic method was the most frequently used for malaria parasite detection (7 studies, 63.6%). In contrast, other studies used RDT (1, 9%)^25^, microscopy/RDT (1, 9%)^12^ for malaria parasite detection, and two studies (2, 18.2%)^26,29^ did not report a method for parasite detection. Five studies (45.5%)^13,14,24,25,27^ used the IMCI definition for pneumonia **(**WHO defined severe pneumonia) and four studies (36.4%)^11,15,16,23^ used chest X-rays for diagnosing severe pneumonia (Table 1). Characteristics of the included studies in detail are demonstrated in Table S2.

**Table 1:** Summary statistics of 11 included studies.

Parameters	Number of study (%)
**Publication years**	
2001–2010	5 (45.5)
2011–2022	6 (54.5)
**Study locations**	
Mozambique	4 (36.4%)
Tanzania	2 (18.2)
Kenya	2 (18.2)
Nigeria	1 (9.1)
Uganda	1 (9.1)
Malawi	1 (9.1)
**Study designs**	
Prospective observational studies	7 (63.6)
Prospective and retrospective observational studies	2 (18.2)
Retrospective observational studies	2 (18.2)
***Plasmodium* co-infected with severe pneumonia**	
*Plasmodium* spp. (did not reported species)	9 (81.8)
*P. falciparum*	2 (18.2)
**Reporting deaths among co-infected patients**	
Yes	5 (45.5)
No	6 (54.5)
**Malaria detection methods**	
Microscopy	7 (63.6)
RDT	1 (9.1)
Microscopy/RDT	1 (9.1)
Not specified	2 (18.2)
**Criteria for severe pneumonia**	
WHO defined severe pneumonia (IMCI definition for pneumonia)	5 (45.5)
Chest X-ray	4 (36.4)
Not specified	2 (18.1)

IFA, immunofluorescence antibody; MAT, microscopic agglutination tests; RDT, rapid diagnostic test; PCR, polymerase chain reaction.

### Quality of the included studies

Based on overall 22 scores, 10 studies (90.9%) were high-quality studies^11,12,16,23–29^; meanwhile, one study was moderate quality^7^. Based on the methodological quality with overall nine scores, 10 studies (90.9%) were high-quality studies^7,11,12,16,23–25,27–29^; meanwhile, one study was of moderate quality^26^. The details of quality assessments are provided in Table S3.

### Proportion of malaria among patients with severe pneumonia

**Figure 2:**
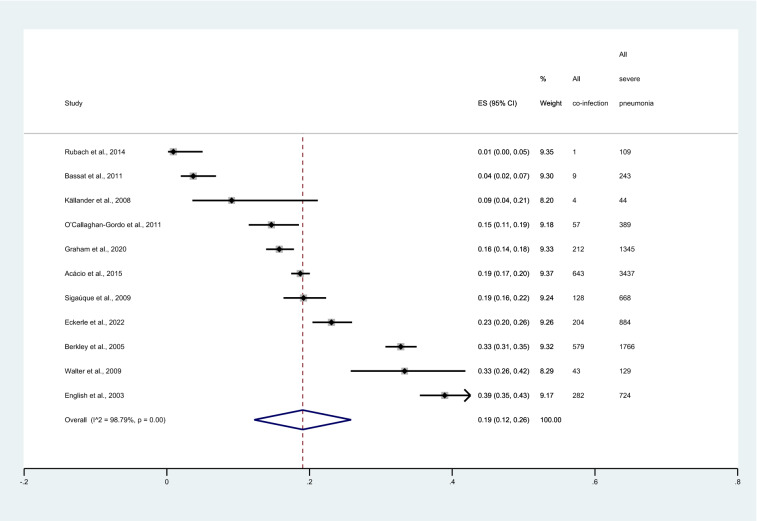
Forest plot demonstrates the pooled proportion of malaria among patients with severe pneumonia. ES, proportion estimate; CI, confidence interval.

The pooled proportion of malaria among patients with severe pneumonia was estimated using the data of 11 studies^7,11,12,16,23–29^. The highest proportion of malaria among patients with severe pneumonia was demonstrated in the study conducted in Kenya during 1999–2000 (39%, 95% CI 35–43%)^7^. Meanwhile, the lowest proportion of malaria among patients with severe pneumonia was demonstrated in the study conducted in Tanzania during 2007–2008 (1%, 95% CI 0–5%)^16^. Overall, meta-analysis results showed that the pooled proportion of malaria (2162 cases) among patients with severe pneumonia (9738 cases) was 19% (95% CI 12–26%, I^2^: 98.79%, 11 studies, Fig. 2).

The meta-regression analysis based on the methodological quality of the studies, publication year, country, study design, age groups, diagnostic methods for malaria, and diagnostic methods for severe pneumonia had been performed to test whether these parameters were source (s) of heterogeneity of the pool proportion of the effect estimates or not. The meta-regression results showed that publication years (P = 0.049) and study design (P = 0.017) were the sources of the heterogeneity of the effect estimates. Meanwhile no statistical difference in the methodological quality of the studies (P = 0.096), diagnostic methods for malaria (P = 0.98), diagnostic methods for severe pneumonia (P = 0.162), country (P = 0.43), and age groups (P = 0.924) in the meta-regression analysis were found (Table 2). The subgroup analysis of publication years (2000–2010 and 2011–2022) and study design (prospective and retrospective observational studies) were performed.

**Table 2:** Meta-regression results.

Parameters	Malaria among severe pneumonia	Severe pneumonia among malaria
Covariates	P-value	P-value
Publication years (2000–2010 vs 2011–2022)	0.049	0.378
Countries	0.43	Insufficient observations
Study designs	0.017	0.378
Age groups (less than 5 years vs less than or more than 5 years)	0.924	0.603
Diagnostic methods for malaria	0.98	0.721
Diagnostic methods for severe pneumonia	0.162	0.916
Methodology quality	0.096	0.93

**Figure 3:**
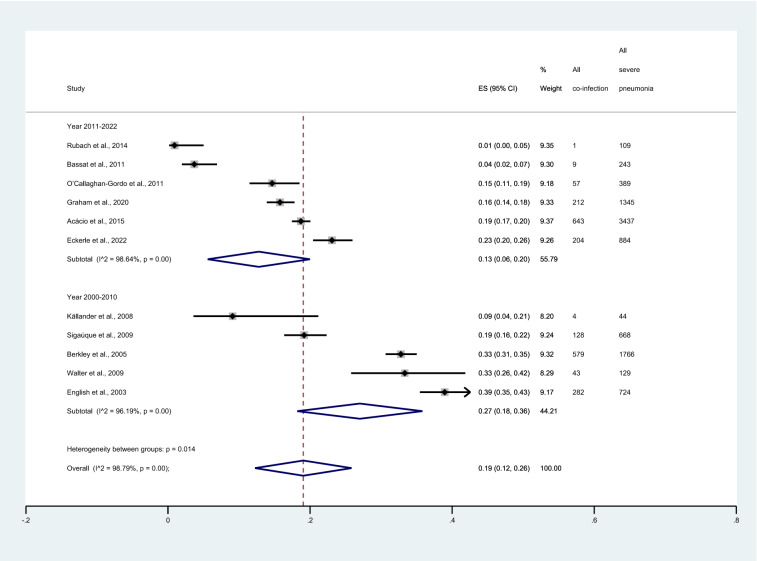
Forest plot demonstrates the pooled proportion of malaria among patients with severe pneumonia sub grouped by publication years. ES, proportion estimate; CI, confidence interval.

**Figure 4:**
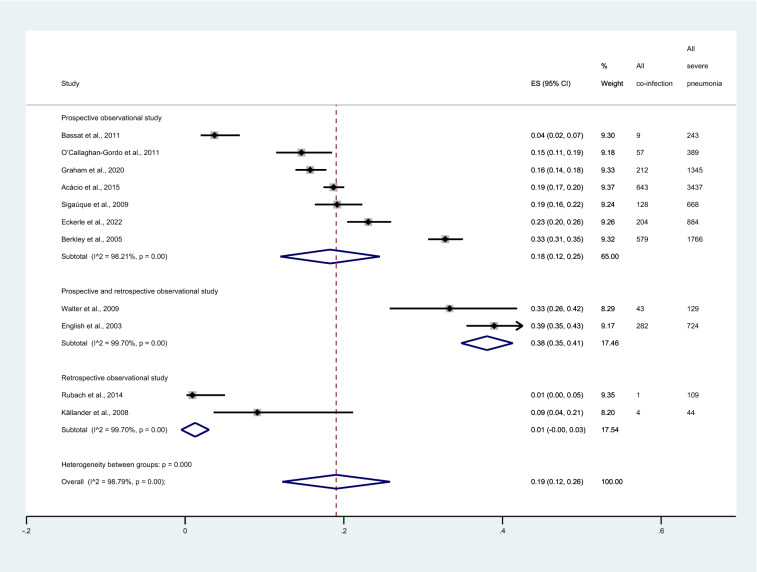
Forest plot demonstrates the pooled proportion of malaria among patients with severe pneumonia sub grouped by study designs. ES, proportion estimate; CI, confidence interval.

Subgroup analysis of publication years showed that the pooled proportion of malaria among patients with severe pneumonia in the year 2011–2022 was 13% (95% CI 6–20%, I^2^: 98.6%, 6 studies). Meanwhile, the pooled proportion among patients with severe pneumonia in the year 2000–2010 was 27% (95% CI 18–36%, I^2^: 96.19%, 5 studies, Fig. 3). Subgroup analysis of study designs showed that the pooled proportion of patients with severe pneumonia among prospective observational studies and retrospective observational studies was 18% (95% CI 12–25%, I^2^: 98.21%, 7 studies), and 1% (95% CI 0–3%, I^2^: 99.7%, 2 studies), respectively. Meanwhile, the pooled proportion of malaria among patients with severe pneumonia among studies that performed both prospective and retrospective observational studies was 38% (95% CI 35–41%, I^2^: 99.7%, 2 studies, Fig. 4).

### Proportion of severe pneumonia among patients with malaria

**Figure 5:**
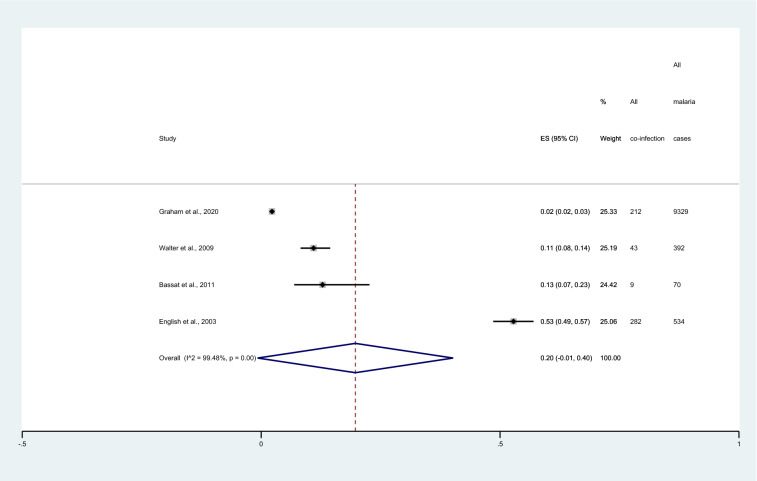
Forest plot demonstrates the pooled proportion of severe pneumonia among patients with malaria. ES, prevalence estimate; CI, confidence interval.

The pooled proportion of severe pneumonia among patients with malaria was estimated using the data of four studies^7,11,12,29^. The highest proportion of severe pneumonia among patients with malaria was 53% (95%CI 49–57%). Meanwhile, the lowest prevalence of severe pneumonia among patients with malaria was 2% (95%CI 2–3%). Overall, the pooled proportion of severe pneumonia (546 cases) among patients with malaria 10,325 cases was 20% (95% CI 0–40%, I^2^: 99.48%, 4 studies, Fig. 5).

The meta-regression results showed no statistical difference in publication years (P = 0.378), and study design (P = 0.378), the methodological quality of the studies (P = 0.93), diagnostic methods for malaria (P = 0.721), diagnostic methods for severe pneumonia (P = 0.916), country (insufficient observations for regression analysis), and age groups (P = 0.603) that affect the pooled estimates (Table 2). The subgroup analysis of these covariates was not further performed.

### Prevalence of deaths among children with co-infection

Four studies^25–28^ reported deaths among patients with malaria and severe pneumonia co-infection. Eckerle et al.^25^ reported that patients with co-infection had a lower proportion of deaths (11/204, 5.39%) than those with severe pneumonia without malaria (10/673, 1.49%). Källander et al.^26^ reported that malaria contributed to 9% (4/44) of pneumonia deaths. O’Callaghan-Gordo et al., reported that patients with co-infection had a comparable proportion of deaths (5/54, 9%) compared to those without malaria (26/300, 9%). Sigaúque et al.^28^ reported that 27% (16/59) of those with malaria and severe pneumonia co-infection led to deaths.

**Figure 6:**
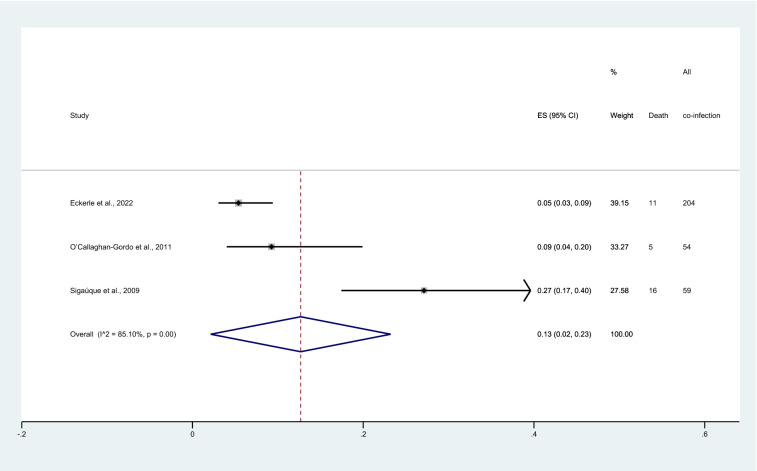
Forest plot demonstrates the pooled proportion of deaths among patients with co-infection. ES, proportion estimate; CI, confidence interval.

The proportion of deaths among patients with co-infection was estimated using the available data from three studies^25,27,28^. The highest proportion of deaths among patients with co-infection was 27% (95% CI 17–40%). Meanwhile, the lowest proportion of deaths among patients with co-infection was 5% (95% CI 3–9%). Overall, meta-analysis results showed that the proportion of deaths among patients with co-infection was 13% (95% CI 2–23%, I^2^: 85.1%, 3 studies, Fig. 6). Källander et al.^26^ reported that all children with co-infection died (4 cases) and were not included in the pooled proportion of deaths. The meta-regression analysis was not performed due to only three studies included in the meta-analysis.

### Sensitivity analysis

**Figure 7:**
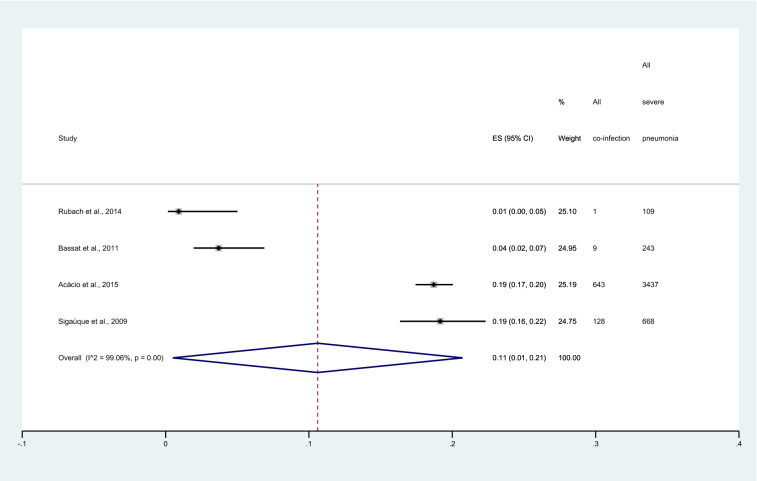
Forest plot demonstrates the pooled proportion of malaria among patients with severe pneumonia in studies that used chest X-ray to confirm the severe pneumonia. ES, proportion estimate; CI, confidence interval.

**Figure 8:**
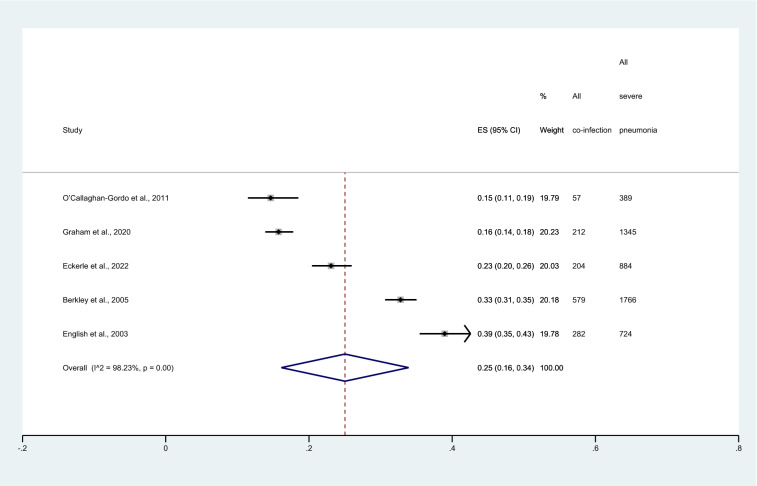
Forest plot demonstrates the pooled proportion of malaria among patients with severe pneumonia in studies that used WHO criteria for pneumonia (IMCI definition) to confirm the severe pneumonia. ES, proportion estimate; CI, confidence interval.

The sensitivity analysis was performed to demonstrate the pooled proportion of malaria and severe pneumonia among studies that used chest X-ray and those that used WHO criteria for severe pneumonia (IMCI definition). Results showed that the pooled proportion of malaria and severe pneumonia among studies that used chest X-rays to confirm severe pneumonia was 11% (95%CI 1–21%, I^2^: 99.06%, 4 studies, Fig. 7). Meanwhile, the pooled proportion of malaria and severe pneumonia among studies that used WHO criteria for severe pneumonia (IMCI definition) to confirm severe pneumonia was 25% (95%CI 16–34%, I^2^: 98.23%, 5 studies, Fig. 8).

## Discussion

The meta-analysis results showed that the pooled proportion of malaria among patients with severe pneumonia was nearly one-fifth and varied according to publication years and study designs. Subgroup analysis of publication years showed that the pooled proportion of malaria among patients with severe pneumonia was highest in 2000–2010 compared to 2011–2022 (27% vs 13%). In addition, subgroup analysis showed the pooled proportion of malaria among patients with severe pneumonia was highest among both prospective and retrospective observational studies (38%). Therefore, the difference in the year of study or the study design might demonstrate the difference in the proportion of malaria among patients with severe pneumonia. In addition, the possible reason for the low proportion of malaria among severe pneumonia might be explained by the fact that patients with co-infection reported by the included studies were not representative of all malaria cases in a malaria-endemic area. For example, in the study by Rubach et al.^16^ that conducted the study in Tanzania where malaria transmission intensity was low. Meanwhile, the high proportion of malaria among patients with severe pneumonia might be explained by the fact that the study was performed in the area where transmission intensity is high such as Kenya^7^. Nevertheless, the meta-regression analysis using country as a covariate did not show statistical significance of the study area to the proportion of malaria among patients with severe pneumonia. There were some other explanations. The diagnosis of malaria cases where only clinical findings were used to diagnose febrile patients might overestimate the proportion of malaria among patients with severe pneumonia. The difference in malaria diagnostic tests might affect the number of malaria cases in different studies. For example, the use of RDT alone instead of microscopy may overestimate the number of malaria cases in the participants as malaria antigen remained in the blood of patients for a period after patients were treated^31^. Nevertheless, the meta-regression using diagnostic method for malaria as a covariate did not show statistical significance of diagnostic method to the proportion of malaria among patients with severe pneumonia. Therefore, there might be some other explanations that remain to be elucidated.

The present study showed that nearly one-fifth of patients with malaria have severe pneumonia. There was a difference in the proportion of severe pneumonia among patients with malaria between studies included in the meta-analysis. The highest proportion of severe pneumonia among patients with malaria (53%) was found in English et al.^7^ that conducted the study in Kenya. Meanwhile, only 2% of patients with malaria have severe pneumonia in Graham et al.^12^ that conducted the study in Nigeria. The possible explanations were the difference in the study sites as all four studies were conducted in different countries of Africa. Nevertheless, based on the limited number of studies, the information was not sufficient for meta-regression analysis using country as a covariate. Furthermore, no probable candidate factor that might explain the difference in the proportion of severe pneumonia among patients with malaria in the meta-regression analysis exists.

The current diagnostic practices of both diseases are based on the clinical findings, chest radiology, culture, and malaria testing, including microscopy/RDT^12^. However, there are some local hospitals where blood cultures and chest X-rays are not performed. Moreover, the only combination of any clinical signs might not be able to reliably distinguish between malaria and severe pneumonia, especially in the absence of chest X‐ray facilities^12^. The laboratory tests for severe pneumonia including blood culture are also exploited as biomarkers to diagnose the type of real etiology in patients such as bacterial or viral severe pneumonia. There are limitations of blood cultures in the diagnosis of childhood pneumonia that the result was only positive in a small number of patients^32^. Therefore, half of the severe pneumonia cases were not given a diagnosis^27,30^, turning results in a high proportion of malaria among severe pneumonia or a low proportion of malaria among patients with severe pneumonia. A previous study showed that positive malaria testing was associated with a lower likelihood of a pneumonia diagnosis, indicating that physicians had a lower underestimation of pneumonia in children without respiratory signs^12^. In addition, patients with malaria admitted to the hospital might not have respiratory symptoms, and malaria incidence was dropped in many African countries^33^, affecting the number of severe pneumonia diagnosed in the hospitals in malaria-endemic countries. There were other factors that affect the proportion of malaria among patients with severe pneumonia or severe pneumonia among patients with malaria. For example, malaria infection caused respiratory complications that lead to pneumonia^34^. In addition, malaria and severe pneumonia co-infections might increase the odds of HIV infection as some included studies enrolled patients with HIV^15,16,26,27^. The impact of HIV on clinical malaria had been exclusively described in the previous systematic review^35–37^. The co-infection of malaria and HIV could lead the patients to severe malaria^35^ and might impact the clinical presentation of severe pneumonia^11^. In addition, HIV-related pneumonia had been reported as a cause of hospital admission^38^.

Results of the meta-analysis showed the difference in the proportion of deaths among patients with malaria and severe pneumonia co-infection. One study^28^ demonstrated 27% deaths among patients with co-infection; meanwhile, one study^25^ demonstrated 5% deaths among patients with co-infection. Sigaúque et al.^28^ which reported the highest proportion of deaths among patients with co-infection at 27% showed that fever and severe anemia were more frequent when clinical malaria and severe pneumonia were present together. In addition, the case fatality rate (CFR) was higher (14%) among co-infection compared with clinical malaria alone (9%)^28^. Eckerle et al.^25^ reported the lowest proportion of deaths among patients with co-infection at 5% showing that children who died were more likely to have malaria and lower hemoglobin than those who survived. The difference in the proportion of deaths between studies might be explained by a difference in the methodology quality among those two studies. Those two studies used different criteria for diagnosing both malaria and severe pneumonia. While Sigaúque et al.^28^ used chest X-rays as a criteria for diagnosing severe pneumonia; Eckerle et al.^25^ used IMCI definitions that lack specificity. Eckerle et al.^25^ used RDT for diagnosing the malaria; meanwhile, Sigaúque et al.^28^ used microscopy which is the gold standard for diagnosing the malaria. Malaria cases might be missed in studies using RDT for diagnosing malaria, and severe pneumonia might be misdiagnosed causing the lower proportion of co-infection and deaths. There has been reported that the poor and missed diagnosis of pneumonia and severe pneumonia, especially in malaria-endemic areas that did not recognize severe pneumonia, has caused respiratory signs in children and is misdiagnosed as severe malaria^13^. This leads to inappropriate treatment of two diseases and can increase the mortality rate. Therefore, a high level of awareness among physician who manage the overlapping of these two infections is required if the diagnostic criteria for both infections is not standardized. The previous study revealed that most children who died from pneumonia were first treated as malaria, which caused delays in seeking healthcare facilities^39,40^.

The present systematic review had limitations. First, as there were a limited number of studies reporting malaria and severe pneumonia co-infection, some of the subgroup analyses contained two or three studies which might affect the robustness of the data. Second, there was high heterogeneity in the pooled prevalence of co-infection between studies. Therefore, a careful interpretation of the pooled estimates was suggested. Third, there was some publication bias caused by the small-study effect. Fourth, some forest plots that present a combined measure with only two studies might reduce the statistical rigor of the present study. Fifth, there was an uncertainty in the diagnosis of severe pneumonia. Some studies used chest X-rays as a diagnostic test for severe pneumonia and some studies did not. Therefore, there were uncertainty in the diagnosis of severe pneumonia.

## Conclusion

In conclusion, nearly one-fifth of patients with severe pneumonia have malaria, one-fifth of patients with malaria have severe pneumonia, and about 13% of co-infections le﻿d to deaths. This information raised the clinical importance of diagnosis and management of concurrent infections. Patients with severe pneumonia should be investigated for malaria, and vice versa. Detection of co-infections might provide the information to inform the physician to manage and cure co-infected patients who live in areas where both diseases are endemic.

## Supplementary Information

Supplementary Table S1.

Supplementary Table S2.

Supplementary Table S3.

Supplementary Information 4.

Supplementary Information 5.

## Data Availability

All data relating to this study are available in the main manuscript and supplementary files.
