# Supplementary material for: Evidence of and deaths from malaria and severe pneumonia co-infections in malaria-endemic areas: a systematic review and meta-analysis
Source: Sci Rep. 2022 Oct 15;12:17344. doi: 10.1038/s41598-022-22151-x (PMC9569341; doi:10.1038/s41598-022-22151-x)
Supplement: Supplementary file 1 — Supplementary Table S1. [file 41598_2022_22151_MOESM1_ESM.docx]

**Evidence of and deaths from malaria and severe pneumonia co-infections in malaria-endemic areas: A systematic review and meta-analysis**

Wanida Mala^1^, Polrat Wilairatana^2^, Giovanni De Jesus Milanez^3^, Frederick Ramirez Masangkay^4^, Kwuntida Uthaisar Kotepui^1^, Manas Kotepui^1*^

^1^Medical Technology, School of Allied Health Sciences, Walailak University, Tha Sala, Nakhon Si Thammarat, Thailand

^2^Department of Clinical Tropical Medicine, Faculty of Tropical Medicine, Mahidol University, Bangkok, Thailand

^3^ Department of Medical Technology, Faculty of Pharmacy, Royal and Pontifical University of Santo Tomas, Manila, Philippines

^4^Department of Medical Technology, Institute of Arts and Sciences, Far Eastern University – Manila, Manila, Philippines

**^*^Corresponding author**

Manas Kotepui; [manas.ko@wu.ac.th](mailto:manas.ko@wu.ac.th), Tel.: +66954392469

Wanida Mala; [wanida.ma@wu.ac.th](mailto:wanida.ma@wu.ac.th)

Polrat Wilairatana; [polrat.wil@mahidol.ac.th](mailto:polrat.wil@mahidol.ac.th)

Giovanni De Jesus Milanez; gmilanez81@gmail.com

Frederick Ramirez Masangkay; frederick_masangkay2002@yahoo.com

Kwuntida Uthaisar Kotepui; [kwuntida.ut@wu.ac.th](mailto:kwuntida.ut@wu.ac.th)

**Table S1. Search term**

| **Databases** | **Search terms/Search strategy** | **Date** |
| --- | --- | --- |
| PubMed | (Malaria OR Plasmodium) AND (Pneumonias OR Pneumonia OR “Lung Inflammation” OR “Lung Inflammations” OR “Pneumonitis” OR “Pneumonitides” OR “Pulmonary Inflammation” OR “Pulmonary Inflammations”) AND (severe OR complicated)  Search option: All fields  Search results: 678 | 21 July 2022 |
| Scopus | (Malaria OR Plasmodium) AND (Pneumonias OR Pneumonia OR “Lung Inflammation” OR “Lung Inflammations” OR “Pneumonitis” OR “Pneumonitides” OR “Pulmonary Inflammation” OR “Pulmonary Inflammations”) AND (severe OR complicated)  Search option: Title, abstract, keywords (as there were too many non-relevant articles retrieved from Scopus if all fields option is used)  Search results: 641 | 21 July 2022 |
| Web of Science | (Malaria OR Plasmodium) AND (Pneumonias OR Pneumonia OR “Lung Inflammation” OR “Lung Inflammations” OR “Pneumonitis” OR “Pneumonitides” OR “Pulmonary Inflammation” OR “Pulmonary Inflammations”) AND (severe OR complicated)  Search option: All fields  Search results: 333 | 21 July 2022 |
| Embase | (Malaria OR Plasmodium) AND (Pneumonias OR Pneumonia OR “Lung Inflammation” OR “Lung Inflammations” OR “Pneumonitis” OR “Pneumonitides” OR “Pulmonary Inflammation” OR “Pulmonary Inflammations”) AND (severe OR complicated)  Search option: All fields  Search results: 997 | 21 July 2022 |
| Ovid | (Malaria OR Plasmodium) AND (Pneumonias OR Pneumonia OR “Lung Inflammation” OR “Lung Inflammations” OR “Pneumonitis” OR “Pneumonitides” OR “Pulmonary Inflammation” OR “Pulmonary Inflammations”) AND (severe OR complicated)  Search option: Search all Ovid journals (abstract only)  Search results: 1137 | 21 July 2022 |
| MEDLINE | (Malaria OR Plasmodium) AND (Pneumonias OR Pneumonia OR “Lung Inflammation” OR “Lung Inflammations” OR “Pneumonitis” OR “Pneumonitides” OR “Pulmonary Inflammation” OR “Pulmonary Inflammations”) AND (severe OR complicated)  Search option: All fields  Search results: 308 | 21 July 2022 |
